# Prospective deployment of an automated implementation solution for artificial intelligence translation to clinical radiation oncology

**DOI:** 10.3389/fonc.2023.1305511

**Published:** 2024-01-04

**Authors:** Christopher E. Kehayias, Yujie Yan, Dennis Bontempi, Sarah Quirk, Danielle S. Bitterman, Jeremy S. Bredfeldt, Hugo J. W. L. Aerts, Raymond H. Mak, Christian V. Guthier

**Affiliations:** ^1^ Department of Radiation Oncology, Brigham and Women’s Hospital, Dana-Farber Cancer Institute, Harvard Medical School, Boston, MA, United States; ^2^ Artificial Intelligence in Medicine (AIM) Program, Mass General Brigham, Harvard Medical School, Boston, MA, United States; ^3^ Radiology and Nuclear Medicine, CARIM & GROW, Maastricht University, Maastricht, Netherlands

**Keywords:** deep learning - artificial intelligence, clinical translation, automated deployment, autosegmentation, quality assurance, end-to-end solution, automated report generation

## Abstract

**Introduction:**

Artificial intelligence (AI)-based technologies embody countless solutions in radiation oncology, yet translation of AI-assisted software tools to actual clinical environments remains unrealized. We present the Deep Learning On-Demand Assistant (DL-ODA), a fully automated, end-to-end clinical platform that enables AI interventions for any disease site featuring an automated model-training pipeline, auto-segmentations, and QA reporting.

**Materials and methods:**

We developed, tested, and prospectively deployed the DL-ODA system at a large university affiliated hospital center. Medical professionals activate the DL-ODA via two pathways (1): On-Demand, used for immediate AI decision support for a patient-specific treatment plan, and (2) Ambient, in which QA is provided for all daily radiotherapy (RT) plans by comparing DL segmentations with manual delineations and calculating the dosimetric impact. To demonstrate the implementation of a new anatomy segmentation, we used the model-training pipeline to generate a breast segmentation model based on a large clinical dataset. Additionally, the contour QA functionality of existing models was assessed using a retrospective cohort of 3,399 lung and 885 spine RT cases. Ambient QA was performed for various disease sites including spine RT and heart for dosimetric sparing.

**Results:**

Successful training of the breast model was completed in less than a day and resulted in clinically viable whole breast contours. For the retrospective analysis, we evaluated manual-versus-AI similarity for the ten most common structures. The DL-ODA detected high similarities in heart, lung, liver, and kidney delineations but lower for esophagus, trachea, stomach, and small bowel due largely to incomplete manual contouring. The deployed Ambient QAs for heart and spine sites have prospectively processed over 2,500 cases and 230 cases over 9 months and 5 months, respectively, automatically alerting the RT personnel.

**Discussion:**

The DL-ODA capabilities in providing universal AI interventions were demonstrated for On-Demand contour QA, DL segmentations, and automated model training, and confirmed successful integration of the system into a large academic radiotherapy department. The novelty of deploying the DL-ODA as a multi-modal, fully automated end-to-end AI clinical implementation solution marks a significant step towards a generalizable framework that leverages AI to improve the efficiency and reliability of RT systems.

## 1 Introduction

Radiation therapy (RT) is increasingly prevalent in cancer treatments with roughly half of cancer patients being prescribed radiation treatment (1, 2). However, numerous factors impede the ability of clinics worldwide to meet the ever-growing demand for RT. As a considerable part of radiation therapy (RT) involves time-consuming manual inputs by health-care professionals with increasing complexity of software, machines and instruments used in daily practice, a significant portion of medical experts’ efforts in radiation oncology is focused on human-machine interactions (2). Further compounding the issue is the lack of well-trained staff as well as treatment facilities, machines, and planning systems, especially in low- and middle-income countries (3).

The large-scale digitization of data in radiation oncology has provided many opportunities for automation tools and applications based on artificial intelligence (AI) (2, 4). Rapid expansion in AI-based technology has given rise to countless new AI computational tools for disease diagnosis (5), medical image segmentation (6, 7), quality assurance (QA) (8), and treatment plan optimization and delivery (9–13). The emergence of deep learning (DL) has enabled significant advances in rapid and reliable automated segmentation platforms for generating accurate contours from routine medical imaging (14–17). Automated medical image segmentation comprises the multifaceted use of image processing techniques, commonly in combination with convolutional neural networks (CNNs) based on the U-Net network architecture (18), which are designed (or “trained”) to identify and label two- or three-dimensional regions of interest corresponding to organs or tumors from magnetic resonance imaging, Computed Tomography (CT) images, or other imaging modalities. Network models extract high- and low-level features from training data with associated ground truth labels wherein weights governing contributions of the various network components are iteratively adjusted via gradient descent to maximize the prediction accuracy of the model for a given set of structures. Clinics and vendors are progressing towards incorporating auto-segmentation tools in the treatment planning workflow to improve the efficiency of organ at risk (OAR) delineation (19–21).

Progress in adapting AI tools into regular clinical use remains slow (22) despite potential to improve the efficiency of radiation oncology clinics (2, 23). Medical software must be evaluated by the Food and Drug Administration (FDA) before entering the US market, an untenably slow process prior to the FDA’s amended regulatory protocol in 2019 (24), and the cost of these systems may also be prohibitive for some less-resourced clinics. Additionally, commercial providers for radiation oncology have been slow to focus efforts on incorporating AI into clinical workflows and their limited access to clinical datasets makes it challenging for them to meet high quality standards (2, 25). Consequently, academic centers are largely left to develop their own home-grown solutions.

In seeking to increase the efficiency of RT patient care, we developed the Deep Learning On-Demand Assistant (DL-ODA), a fully automated, end-to-end AI clinical implementation solution to enable clinical deployment and translational research. This software aims to significantly reduce the time required to perform segmentation as well as background QA to detect errors associated with (1) inaccurate manual contours or (2) insufficient OAR sparing or incorrect dosimetric targeting. The DL-ODA provides immediate and/or ambient (background) AI deployment at individual institutions featuring (1) an automated data processing, model-training and validation pipeline, and (2) a clinical deployment route allowing models to be directly deployed in the clinic for segmentation and QA purposes. Here, we demonstrate the efficacy of our system based on both internally and externally trained models.

## 2 Materials and methods

**Figure 1:**
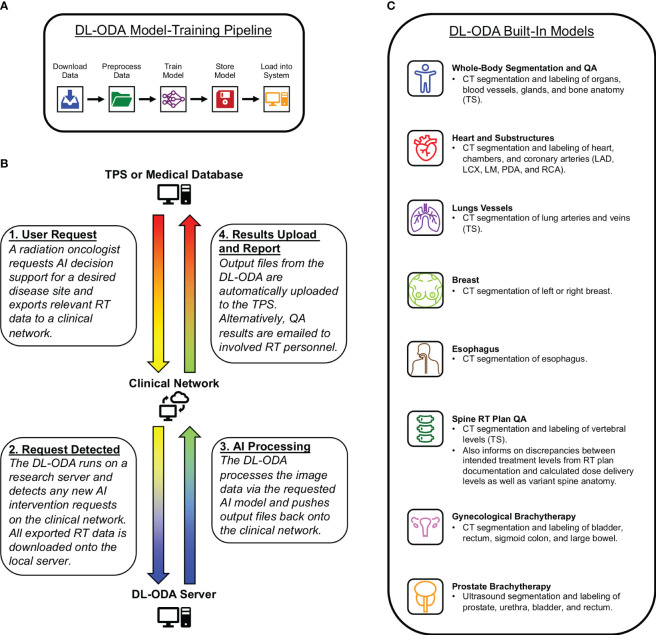
**(A)** A medical professional activates an auto-segmentation and/or QA request for a desired disease site by exporting relevant RT data to a clinical network from a radiation oncology TPS or RT database. The DL-ODA downloads the exported RT data from the clinical network onto a research workstation and begins processing using the desired AI model. Once finished, any output files generated by the DL-ODA are uploaded back onto the network drive where they are subsequently uploaded to the Eclipse server. Alternatively, QA results are emailed as a report to appropriate clinical personnel. **(B)** Overview of our built-in automated DL model-training pipeline with access to available clinical datasets. **(C)** The various AI models integrated into the DL-ODA.

The DL-ODA features (1): automated DL model-training pipeline (**Figure 1A**) (2); clinical integration with a given institutional treatment planning system (TPS) and/or electronic medical record (EMR) system, allowing comparison of clinical contours against DL contours and automated dose-volume histogram (DVH) calculation of approved clinical RT plans on DL contours (**Figure 1B**) (3); automated clinical deployment (4); pre-integrated algorithms drawing from publicly available frameworks and available RT specific auto-segmentation models from our institution and beyond (**Figure 1C**); and (5) automated reporting of QA results, including assessments of manually delineated OARs against AI prediction masks (contour QA) and dose target verification of treatment plans (dose delivery QA).

The DL-ODA establishes two deployment pathways for clinical use (1): the On-Demand pathway and (2) the Ambient pathway. The On-Demand pathway allows immediate AI decision support for a clinician for a specific disease site. The Ambient pathway includes AI interventions at scheduled times for continuous QA of any RT plans. Both pathways utilize publicly available models selected for relevance to RT treatment planning as well as models developed within our institution (26, 27). For On-Demand, a user requests AI assistance by exporting relevant RT data to a specified location on a clinical network. The DL-ODA, which runs on a Linux-based research server, periodically listens for new requests on the network. Once a request is detected, the DL-ODA downloads the relevant RT data files (including image, structure set, and/or RT plan data) onto the server and performs the auto-segmentation and/or QA tasks indicated by the user via the appropriate DL algorithms for the given disease site. DL segmentation outputs can be pushed back onto the clinical network where they are automatically uploaded onto the TPS. Additionally, a summary of QA results can be emailed to the appropriate clinical personnel. As all tasks are completed fully within the scope of the clinical network, the data are protected by the institutional firewall which absolves the need for additional deidentification of patient sensitive information. For fast deployment, DL-ODA has been designed with an interface that allows exporting image data using a binary format which exports only the raw pixel data and geometrical information. For this mode, no patient information leaves the treatment planning system.

We developed, tested, and prospectively deployed DL-ODA at our institution’s RT clinic. Medical professionals activate a request for the DL-ODA through (1): a TPS while viewing a given patient image scan for the desired disease site, in which DL-ODA acquires relevant image data and returns DL segmentations to be automatically uploaded back to the TPS for immediate visualization, or (2) a radiation oncology clinical informatics database or EMR, in which RT planning data are downloaded and processed in order to perform a specified QA task. All auto-segmentation tasks are processed using an NVIDIA RTX A4000 graphics processing unit. The methodology and implementation are simple and can be adopted for any RT clinic using our open-source deployment scripts accessible via ModelHub.ai (https://mhub.ai/models/DL-ODA).

### 2.1 Model-training pipeline to facilitate end-to-end training to deployment

We implemented a fully automated pipeline model-training pipeline for clinical datasets (**Figure 1A**). Image and structure set data are downloaded, preprocessed, and converted into a format compatible with the nnU-Net framework. Resulting model segmentations are postprocessed and validated based on DSC or other metrics. Both preprocessing and postprocessing routines are customizable, allowing users to set desired thresholds for windowing, image cropping, and normalization. This model-training pipeline is ideal for large clinical datasets, but automatic image augmentation based on translation, rotation, dilation, and noise generation is also available for smaller datasets. Our institutional models were trained with two NVIDIA RTX A6000 GPUs using either TensorFlow (version 2.10.1) or PyTorch (version 2.0.1) frameworks and the Python programming language (version 3.9.12) for scripting. Once training is completed, the model performance is automatically evaluated by comparing AI prediction volumes from the test set against ground truth segmentations using metrics such as volumetric Dice coefficient (DSC) (28), Hausdorff distance (HD) (29), average symmetric surface distance (ASSD) (30), and relative volume difference (30). The median and interquartile ranges for test performance distributions are exported along with box plots for user review. Additionally, we perform dosimetric validation by calculating and comparing DVHs for all structures.

### 2.2 On-demand deployment pathway for efficiency, research or real-time QA

**Figure 2:**
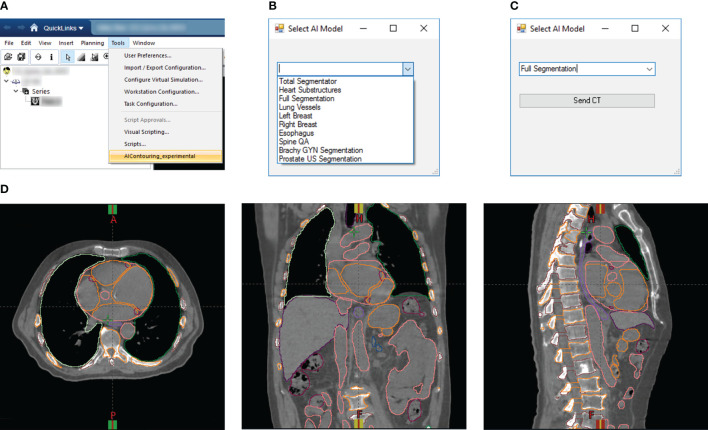
**(A)** Screenshot of Eclipse treatment planning interface showing access to our custom Eclipse API which activates the DL-ODA. **(B)** Once activated, the DL-ODA allows a user to choose from a selection of built-in DL models. **(C)** The relevant patient image data can be exported for processing with the chosen model. **(D)** The resulting DL segmentations are automatically uploaded into the Eclipse treatment planning system for visualization. Shown are axial (left), coronal (middle), and sagittal (right) views for an example treatment planning case with auto-segmentations via the Full Segmentation model consisting of whole-body segmentations (TotalSegmentator) and heart substructures segmentations with coronary arteries visible.

For rapid DL segmentation and contour visualization, we achieve DL-ODA activation through our Departmental TPS (Eclipse, Siemens Healthineers, Erlangen, Germany) using our custom Application Programming Interface (API) script written in the C# programming language (**Figures 2A–C**). The user selects the requested DL model from a drop-down list and exports the appropriate image data to a location on a clinical network drive. For dose delivery QA, dose distribution data is also exported to allow calculation of dose metrics of our choosing. This routine supports two export formats (1): Digital Imaging and Communications in Medicine (DICOM), and (2) our custom binary format which filters the export data down to only the essential components (image volume data, voxel spacing, orientation, and space origin) to reduce data transfer and computation time. Both formats include information about the Series Instance UID to uniquely identify the given treatment plan as well as the desired DL model information embedded in the output folder and file names. The DL-ODA detects any new patient image data outputs from the user’s Eclipse server on the network drive and downloads the exported data to the research workstation to begin processing and model deployment. When finished, the DL segmentations are uploaded back onto the clinical network and the Eclipse API uploads them into the TPS for immediate contour visualization (**Figure 2D**). The DL-ODA handles multiple simultaneous requests by processing each request sequentially on a first-come first-served basis.

### 2.3 Ambient deployment pathway for QA applications and reporting

The QA tasks are accomplished by exporting desired RT data (including image, dose, plan, and/or structure set data) to the clinical network in DICOM format via SQL DICOM query retrieve from the Departmental clinical database and radiation oncology EMR (ARIA, Varian Medical Systems). The DL-ODA pulls this data onto the research workstation for processing. Common to all models is the contour QA which evaluates existing manual segmentations against DL segmentations for the same structure. The DSC is computed for each pair of manual and matching DL segmentations, using the entire manual and DL volumes for comparison (Full DSC). A separate DSC is computed based on manual and DL sub-volumes contained within any axial slices that were manually contoured (Partial DSC). The latter accounts for cases where clinicians only contoured the portion of a given structure deemed sufficient in representing clinically significant dosimetric consequences (such as partial esophagus) as this would otherwise provide a reduced Full DSC value. The DL-ODA automatically generates an email report including results for all matched pairs to clinical end-users. As with the On-Demand pathway, the dose delivery QA for the Ambient pathway involves calculation of selected dose metrics pertaining a given treatment plan based on the overlap of the dose distribution with the contoured structures.

### 2.4 Pre-loaded auto-segmentation models

To improve the cost effectiveness and accessibility of AI implementation in a given department’s clinic, the DL-ODA system was specifically designed to be used with both publicly available models and models developed within our own institution. This section summarizes all pre-loaded auto-segmentation models which include publicly available models, institutional models trained prior to the development of DL-ODA, and models trained using the aforementioned DL-ODA end-to-end model-training pipeline.

#### 2.4.1 Publicly available model – TotalSegmentator

The DL-ODA utilizes whole-body DL segmentations provided by TotalSegmentator (31), a publicly available medical image segmentation tool based on the nnU-Net framework (32) that can generate over 100 different anatomical structures from CT imaging, including gastrointestinal organs (esophagus, stomach, duodenum, small bowel, large bowel, and bladder), cardiovascular structures (heart chambers, aorta, pulmonary artery, portal vein and splenic vein, inferior vena cava, iliac artery, and iliac veins), cervical and thoracic structures (brain, face, trachea, bronchi, lung lobes, lung vessels, adrenal glands, spleen, liver, gallbladder, pancreas, and kidneys), skeleton (clavicles, humeri, scapulae, hip bones, femurs, ribs, vertebrae and sacrum), and muscles (autochthons, iliopsoases, and gluteal muscles).

#### 2.4.2 Institutional previously trained models

The following are institutional AI models created before the development of DL-ODA that were incorporated into the system post-facto. These models are primarily based on CT image segmentation. However, DL-ODA can be used for other imaging modalities as well. We have successfully trained and validated a GU model transrectal US based images acquired during HDR brachytherapy. We further developed a GYN model for our MRI guided brachytherapy program allowing contour propagation and dose assessments during treatments.

##### 2.4.2.1 Gynecological brachytherapy

Our custom Gynecological Brachytherapy model assists with treatment planning patients undergoing interstitial brachytherapy for the cervix. The model quickly contours daily anatomy (including bladder, rectum, sigmoid colon, and small bowel) and evaluates dose to OARs for verification. Model training was performed using the nnU-Net framework.

##### 2.4.2.2 Prostate brachytherapy

We trained a custom model for segmenting critical OARs from ultrasound images related to prostate brachytherapy. Prostate, urethra, bladder, and rectum segmentations are rapidly generated. Robust model performance was verified based on a testing subset of prostate brachytherapy cases independent of training (33).

##### 2.4.2.3 Whole heart

A whole-heart model trained using radiologically-acquired ground truth labels was implemented and evaluated based on a clinical trial involving an independent real-world dataset of 5677 breast cancer patients treated with radiation therapy at the Dana-Farber/Brigham and Women’s Cancer Center without retraining the model (27, 34). The resulting DL heart segmentations are highly accurate and demonstrate a successful transfer of learning to this real-world clinical dataset.

#### 2.4.3 Models trained using the model-training pipeline

The following models were trained with the DL-ODA model-training pipeline drawing from existing clinical datasets.

##### 2.4.3.1 Esophagus

We trained a model for whole esophageal segmentation and incorporated into the DL-ODA to provide reference AI contours for clinicians who desire quick confirmation for this structure that is both challenging to delineate (35) and prone to segmentation variations between individuals (36). Training was achieved based on a cohort of 394 patients treated at our institution predominantly for lung cancer using a modified U-Net architecture via TensorFlow. This model is currently used to detect inconsistencies and variations in esophagus delineations between different observers in treatment planning.

##### 2.4.3.2 Heart and heart substructures

For particular applications where sparing of the heart and coronary arteries are critical (for example, in lung RT), we trained a collection of heart segmentation models for the heart (34) and heart substructures, including left and right atria, left and right ventricles, and five coronary arteries including the left main, left anterior descending, left circumflex, right, and posterior descending arteries (LM, LAD, LCX, RCA, and PDA, respectively) (26, 37). Separate models were trained for all structures based on a multi-institutional cohort of 699 CTs image datasets with manually contoured heart and substructure masks. Final training of each model was achieved using nnU-Net.

### 2.5 Clinical model deployment and testing

Prospective clinical deployment and testing involved assessment of auto-segmentation and QA performances of the various requestable AI interventions available through the DL-ODA system.

#### 2.5.1 Whole-body segmentation, full segmentation, and QA

The whole-body segmentation is based on TotalSegmentator. Full segmentation involves the combination of whole-body segmentation via TotalSegmentator and the Heart Substructures model. Though TotalSegmentator already provides segmentations for the chambers of the heart, these are replaced with the more accurate Heart Substructures segmentations (**Figure 2D**). Contour QA is available for both pathways and involves the evaluation of existing manual segmentations against DL segmentations from TotalSegmentator (provided the given structure is included as one of the DL outputs) with results emailed to end-users.

#### 2.5.2 End-to-end model training and deployment for breast segmentation

In 2023, our institution clinically deployed a vendor-implemented DL algorithm designed to provide a baseline set of contours that are readily editable by radiation oncology staff. Numerous clinical breast specialists within our department expressed a particular desire for AI breast segmentations that more closely reflect established clinical practice compared with those generated by the vendor algorithm. This prompted us to use the model-training pipeline built into the DL-ODA to train left and right breast segmentation models optimized to our clinicians’ practice. To assess the efficiency of our model-training pipeline, we timed the entire workflow from start to finish, including procuring and exporting the training data, downsampling the image and label volumes, training all models, upsampling prediction outputs for validation, and deploying the finalized models into the clinic. Model training was achieved via the nnU-Net framework. These breast segmentations are currently undergoing clinical evaluation.

#### 2.5.3 Retrospective evaluation of contours for QA

The contour QA functionality of the DL-ODA was tested based on a retrospective cohort of 4,284 RT treatment plans drawn from 3,399 lung and 885 spine treatments procured from a RT database representing a large, multi-center department that treats roughly 5,000 patients with RT annually. For each case, CT image data were auto-segmented using our Full Segmentation routine (TotalSegmentator and our Heart and Heart Substructures model) while manual contours were acquired for comparison. Full DSC and Partial DSC metrics were computed for manual-DL segmentation pairs automatically detected by comparing the structure labeling nomenclature, based on the American Association of Physicists in Medicine Task Group 263 (38), to known TotalSegmentator output labels. In addition, this allows us to analyze plan quality and evaluate dosimetric trends for individual structures. Currently, our department uses a DSC score of 0.85 as a threshold for passing geometric agreement (34). For future work, we envision relying on dose metrics to automatically trigger a detailed review if a discrepancy in prescribed metrics between the clinical and the DL-ODA structures is discovered.

#### 2.5.4 QA for dose delivery for approved clinical RT plans

The DL-ODA features automated DVH calculation for all DL-generated structures using dose distribution data acquired from approved clinical RT plans (39). This allows for verification of dosimetric coverage and targeting if desired. Currently, this feature is enabled for spine RT treatment plans and RT plans for which dosimetric sparing of the heart and coronary arteries are critical (such as lung RT). The results of both the spine and heart applications are currently being prepared for publication.

Spine RT treatment planning is prone to wrong-anatomic-level treatment errors resulting from incorrect enumeration due to morphology with only partial imaging of the spine and occurrence of variant spine anatomy (40–42). The DL-ODA can provide vertebral segmentations from TotalSegmentator as quick confirmation of vertebral contours and to help prevent labeling errors. DL vertebral segmentations (with associated labels) that are uploaded onto a TPS serve as a visual reference for clinicians as they verify dosimetric targeting. Alternatively, DVH calculations for each individual DL vertebrae facilitates identification of target levels which are compared with documented levels in the RT prescription. The results are emailed to the appropriate clinical personnel.

For dose delivery QA for the heart, we use DL segmentations generated from our own Whole Heart and Heart Substructures models. As with the spine RT plan QA, output DL segmentations from the Heart Substructures or the Whole Heart models can either be uploaded to the TPS for immediate viewing or be used in DVH calculations. For the latter case, the DL-ODA generates a report containing contour QA for each structure as well as dose metrics for heart and coronary arteries. A summary of the results is emailed to the appropriate clinical personnel.

#### 2.5.5 Segmentation of lung vessels for research

To demonstrate an example of utilizing DL-ODA as a useful platform for research, we implemented a Lung Vessels segmentation pathway with the prospect that dose to these structures may be used to predict patient survivability. The Lung Vessels model is an extension of whole-body segmentation in which pulmonary vasculature is generated along with the lungs, trachea, and bronchi. The goal is to provide dosimetric measures for those structures during lung RT.

### 2.6 Prospective clinical deployment of DL-ODA

In addition to retrospective testing, the DL-ODA has been prospectively deployed for clinical use in our institution’s treatment planning system. All ambient QA emails generated following deployment have been carefully examined manually.

## 3 Results

### 3.1 Evaluation of end-to-end training and deployment of breast models

**Figure 3:**
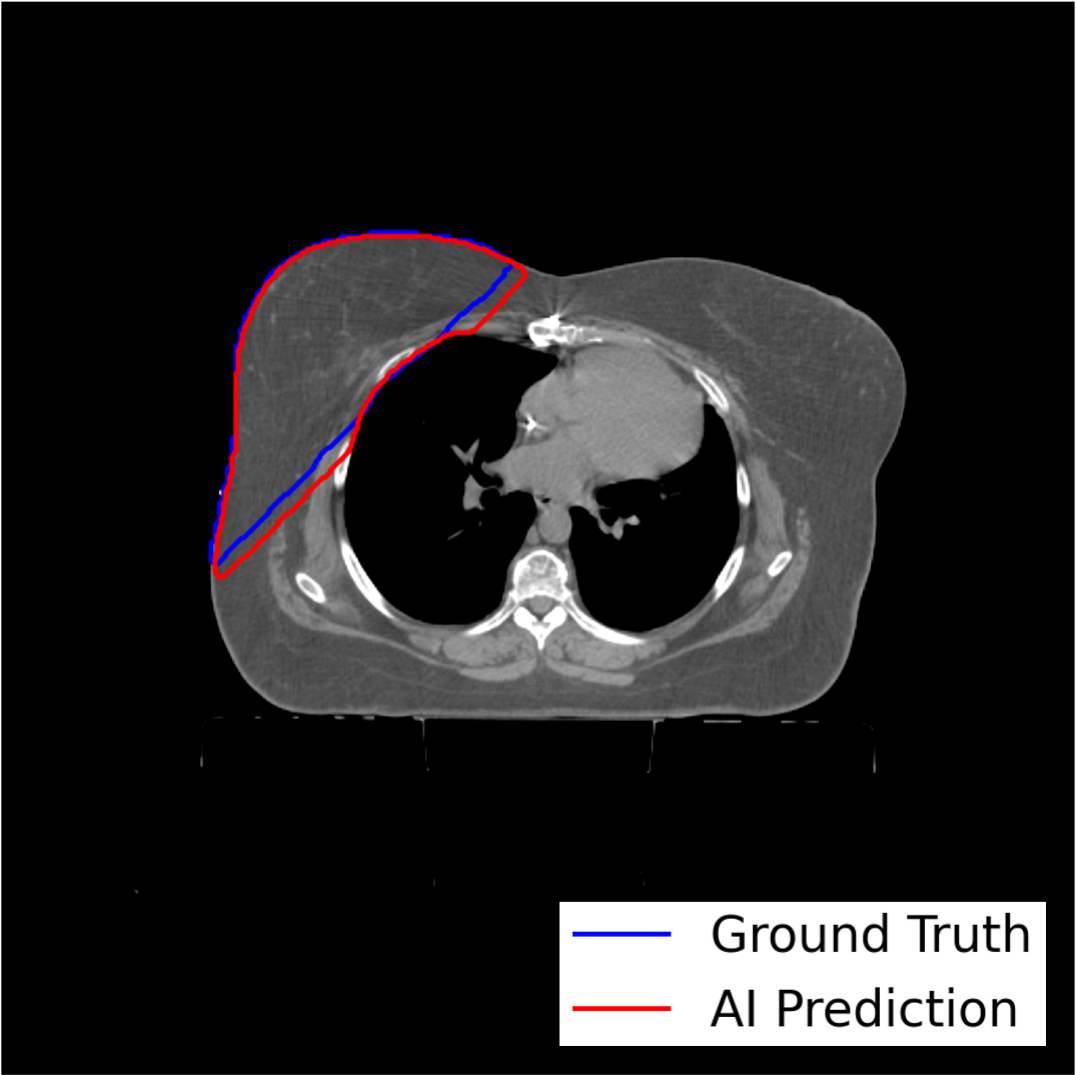
Axial image output for a representative test breast RT case demonstrating high similarity between the expertly contoured ground truth label for the right breast and the prediction label generated by the right breast segmentation model trained using the model-training pipeline.

The model-training pipeline successfully trained left and right breast models using a dataset of 1,823 breast RT cases (935 for left breast and 888 for right) within 9 hours and 21 minutes and retrieved all training data from the clinical database in 46 hours. Both models were validated based on testing subsets with prediction label volumes closely matching ground truth delineations contoured by clinical experts (**Figure 3**), thus confirming that the pipeline successfully enabled breast segmentations that are clinically desirable. Comparisons between ground truth and AI prediction segmentations were evaluated using DSC, HD, ASSD, and VD (**Table 1**). The breast segmentation models were also evaluated dosimetrically based on quantifying differences of D_max_ and D_mean_ between ground truth and AI prediction volumes (**Table 2**). The results of both geometric and dosimetric evaluations yielded close agreement between AI- and manually generated structures.

**Table 1:** Ground truth versus AI segmentations were geometrically evaluated for 183 breast contours from the test sets of the breast segmentation models (94 for left and 89 for right breast).

Structure	Number of Cases	Median Full ΔDSC (IQR)	Median Partial ΔDSC (IQR)	Median Full ΔHD (IQR) (mm)	Median Partial ΔHD (IQR) (mm)	Median Full ΔASSD (IQR) (mm)	Median Partial ΔASSD (IQR) (mm)	Median Full ΔVD (IQR) (cm^3^)	Median Partial ΔVD (IQR) (cm^3^)
Breast Segmentation Results									
Left Breast	94	0.91 (0.87 - 0.93)	0.91 (0.87 - 0.93)	19.7 (13.2 – 26.4)	18.4 (13.3 – 24.9)	2.83 (2.09 – 3.47)	2.70 (1.81 – 3.29)	156 (58.8 – 302)	156 (59.5 – 302)
Right Breast	89	0.92 (0.89 - 0.94)	0.92 (0.89 - 0.94)	19.6 (12.0 – 25.5)	19.7 (16.0 – 27.3)	2.74 (2.14 – 3.51)	2.80 (2.03 – 3.58)	145 (60.5 – 264)	145 (70.7 – 264)
Contour QA Results									
Heart	3,258	0.91 (0.87 - 0.93)	0.94 (0.92 - 0.95)	13.8 (10.7 – 20.0)	12.0 (9.52 – 16.1)	3.29 (2.42 – 4.81)	1.77 (1.35 – 2.50)	85.9 (43.5 – 159)	44.1 (19.2 – 83.3)
Left Lung	2,159	0.96 (0.95 - 0.97)	0.96 (0.95 - 0.97)	16.4 (12.0 – 25.5)	15.8 (11.5 – 23.8)	1.17 (0.897 – 1.64)	1.14 (0.887 – 1.61)	62.3 (33.3 – 108)	61.4 (32.4 – 107)
Right Lung	2,169	0.97 (0.95 - 0.97)	0.97 (0.95 - 0.97)	15.8 (12.0 – 23.8)	15.0 (11.5 – 21.8)	1.32 (0.986 – 1.84)	1.30 (0.971 – 1.80)	65.6 (28.7 – 117)	63.7 (27.8 – 114)
Esophagus	2,893	0.76 (0.59 - 0.81)	0.81 (0.76 - 0.84)	18.8 (11.3 – 62.2)	10.5 (7.57 – 15.5)	1.78 (1.24 – 7.07)	1.17 (0.925 – 1.52)	7.75 (3.30 – 16.3)	3.58 (1.54 – 6.98)
Trachea	394	0.57 (0.51 - 0.63)	0.73 (0.67 - 0.79)	47.8 (39.6 – 55.8)	7.38 (5.47 – 11.6)	7.44 (5.64 – 9.16)	2.08 (1.53 – 2.57)	6.34 (3.01 – 12.2)	11.4 (6.97 – 16.7)
Liver	367	0.94 (0.93 - 0.95)	0.95 (0.93 - 0.96)	20.0 (15.1 – 30.6)	18.2 (14.5 – 24.9)	1.79 (1.49 – 2.51)	1.70 (1.40 – 2.18)	60.3 (27.8 – 115)	48.9 (22.1 – 89.0)
Stomach	216	0.74 (0.53 - 0.85)	0.86 (0.79 - 0.90)	51.5 (28.6 – 86.9)	22.6 (13.6 – 38.4)	5.99 (2.68 – 12.3)	2.11 (1.47 – 3.36)	60.0 (23.3 – 136)	22.7 (8.31 – 49.3)
Small Bowel	134	0.46 (0.26 - 0.60)	0.70 (0.55 - 0.80)	116 (80.0 – 149)	57.5 (37.9 – 75.3)	17.2 (8.78 – 31.1)	4.74 (2.71 – 9.41)	390 (233 – 533)	71.2 (25.3 – 191)
Left Kidney	363	0.91 (0.88 - 0.93)	0.91 (0.88 - 0.93)	10.2 (7.65 – 13.8)	9.88 (7.48 – 13.1)	1.63 (1.13 – 2.27)	1.57 (1.07 – 2.13)	17.1 (6.83 – 30.8)	16.2 (6.54 – 29.1)
Right Kidney	364	0.91 (0.88 - 0.93)	0.91 (0.88 - 0.93)	10.5 (7.81 – 13.7)	10.1 (7.58 – 13.3)	1.61 (1.17 – 2.19)	1.54 (1.12 – 2.12)	15.1 (6.55 – 28.8)	15.1 (6.29 – 28.3)

Additionally, manual-DL segmentation pairs were evaluated for ten organs-at-risk (OARs) that were contoured for greater than 100 cases out of 4,284 total for the retrospective contour QA. Geometric distributions were acquired via pairwise subtraction of Dice similarity coefficient (DSC), Hausdorff distance (HD), average symmetric surface distance (ASSD), and volume difference (VD) for each patient. Median values and interquartile ranges (IQRs) are reported for each distribution of metric differences (ΔDSC, ΔHD, ΔASSD, ΔVD) based on full ground truth and AI segmentations as well as partial segmentations which include only portions of structures that appear on axial slices which were manually contoured.

**Table 2:** Dosimetric distributions were acquired via pairwise subtraction of D_max_, V100, and V105 of AI full breast segmentations from those of the ground truth volumes for each of 183 test patients from the breast segmentation model training datasets.

Median ΔD_mean_ (IQR) (cGy)	Median ΔV100 (IQR) (%)	Median ΔV105 (IQR) (%)
-50.6 (-111 – -1.40)	-3.5 (-8.0 – 0.0)	0.0 (-1.0 – 0.0)

The median and IQR are reported for each distribution of metric differences (ΔD_max_, ΔV100, and ΔV105).

### 3.2 Evaluating on-demand contour QA application

**Figure 4:**
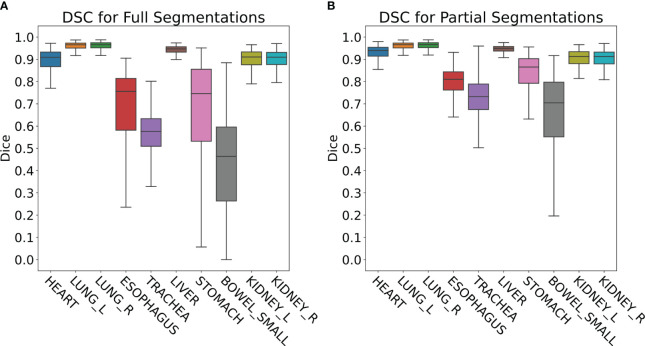
**(A)** Full DSC distributions for the ten organs-at-risk (OARs) listed in **Table 1**. Generally close agreement between manual and DL segmentations is observed for heart, lungs, liver, and kidneys but is significantly lower for esophagus, trachea, stomach, and small bowel. **(B)** Partial DSC distributions for the same ten OARs. Improved similarity is observed in all cases. This is especially true for esophagus, trachea, stomach, and small bowel, possibly indicating prevalence of incomplete or inaccurate contouring of these structures by clinicians.

The retrospective contour QA study revealed ten most common organs-at-risk (OARs) that were contoured for greater than 100 cases out of the 4,284 total cases (**Table 1**). Median Full DSC metrics exceeded 0.9 for heart, left and right lungs, liver, and left and right kidneys (**Figure 4A**), indicating that model segmentations for lungs, liver, and kidneys are in close agreement with those provided by clinical experts. The heart segmentations from our Heart Substructures model are also highly consistent with manual delineations. Lower median Full DSC was exhibited by esophagus (0.76), trachea (0.57), stomach (0.74), and small bowel (0.46). However, median partial DSC metrics were markedly improved for trachea (0.73), stomach (0.86), and small bowel (0.70) with lesser improvement for esophagus (0.81), possibly indicating prevalence of incomplete or inaccurate manual contouring of these structures by clinicians (**Figure 4B**). Similar trends between full and partial segmentation comparisons are also observed for HD, ASSD, and VD. The average time required to process a given RT plan across all models was roughly ten minutes.

### 3.3 Clinical deployment of ambient QA email reports

**Figure 5:**
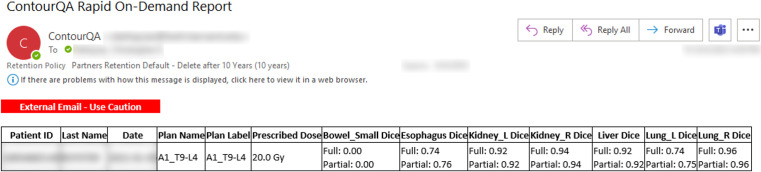
Screenshot of an example email report issued by the contour QA functionality built into the DL-ODA. The results shown are for a spine RT treatment plan. The low DSC values for small bowel would likely necessitate manual review of the manual delineation for this structure.

**Figure 6:**
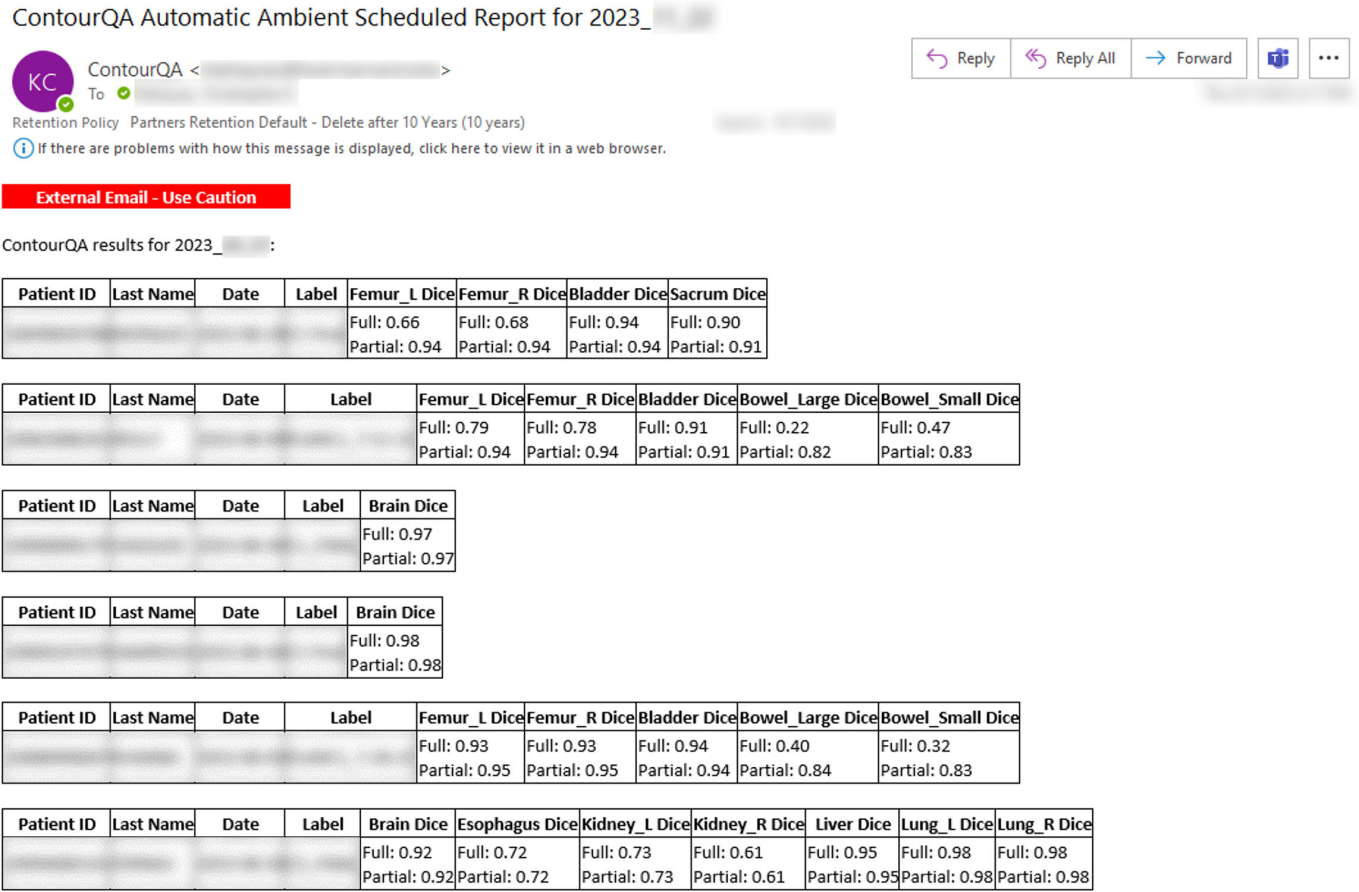
Screenshot of an example email report issued by the Ambient Contour QA from the DL-ODA system for all treatment plans created on a given day. This collection of treatment plans features a variety of disease sites.

All QA results processed by the DL-ODA system were successfully generated and emailed to appropriate RT personnel. On-Demand contour QA reports for individual treatment plans included information about the patient, RT plan, prescription dose, and Full DSC as well as Partial DSC for any detected manual-DL segmentation pairs (**Figure 5**). Ambient contour QA reported the same information included in On-Demand contour QA reports but for a collection of all treatment plans for the given day (**Figure 6**).

Ambient QA for Heart Substructures includes information about the patient, RT plan, prescription dose, and contour QA for whole heart. Additional dose metrics such as mean dose to the heart and coronary arteries are also provided. All results are emailed as a report to the appropriate personnel. This Ambient QA has processed over 2,500 treatment plans over the past 9 months. For Ambient spine RT QA, verification of dosimetric targeting is relayed to clinical staff via email reports which includes alerts for any targets that overdosed or underdosed. The report also raises alerts for any vertebral geometric inconsistencies that are detected based on the DL spine segmentations, indicating potential variant spine anatomy or presence of other anomalous factors (e.g. hardware or implants, sacralization, or collapsed vertebrae). Generated email reports include information about the patient, RT plan, intended clinical target levels, and dose prescription. The Ambient QA for spine RT has processed over 230 treatment plans over the past 5 months.

### 3.4 Dosimetric quantification of lung vessels

**Figure 7:**
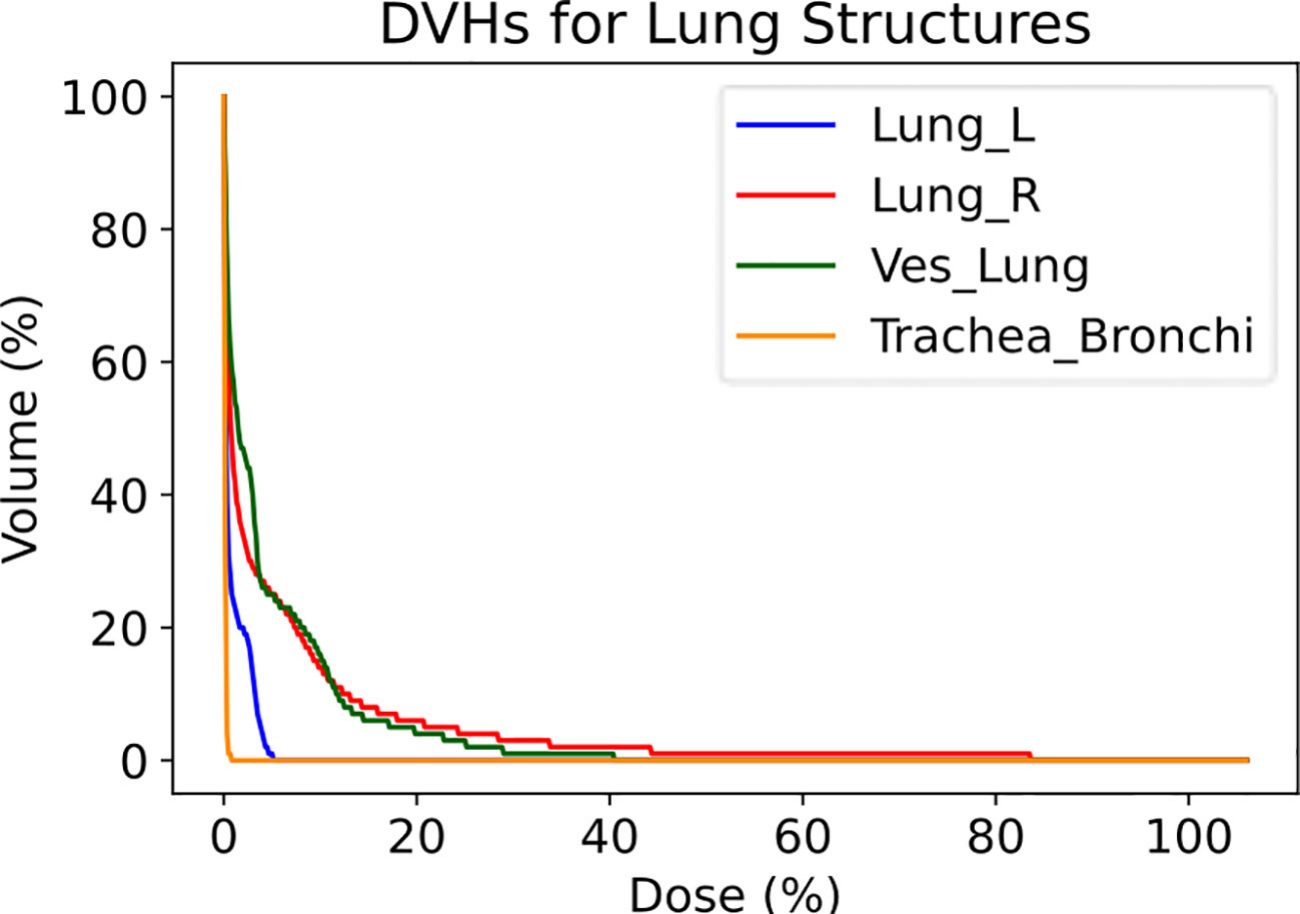
Dose-volume histograms (DVHs) for a lung RT case treated using VMAT with standard fractionation for confirmation of dosimetric consequences to relevant critical structures. The left lung, right lung, lung vessels, and combined trachea and bronchi structures were auto-segmented using TotalSegmentator.

The Lung Vessels model successfully segmented vasculature in left and right lungs and exported DVHs for both lungs, lung vessels, and combined trachea and bronchi structures, thus informing on dosimetric trends usable in research purposes (**Figure 7**).

## 4 Discussion

The DL-ODA system is capable of drastically enhancing clinicians’ RT planning experience by providing automated segmentations for various disease sites as well as feedback for contour delineations and dosimetric targeting or coverage. The system thus serves as an efficient aid for clinical staff by reducing the time required to perform manual delineations or QA checks. The implementation of DL-ODA is simple and based on zero-cost software components including the Python programming language, TensorFlow, TotalSegmentator, and a Linux-based operating system, rendering customization towards a particular clinical institution’s needs an easy task. It is currently running on one of our lab computers, though migration to a more dedicated server is planned for the near future. The novelty of deploying DL-ODA not only as an auto-segmentation tool but also as an automated QA system is particularly advantageous. Given the rising complexity of RT treatment planning that is correlated with an increased use of ancillary devices and new treatment techniques, automation systems are crucial for reducing RT inaccuracies due to manual contouring, data entry, and setups (43).

Our model-training pipeline successfully generated new DL algorithms for breast segmentation in an exceptionally efficient timespan. Furthermore, the test prediction segmentations of the trained breast models closely resembled the breast delineations provided by clinical experts, confirming the immediate utility of the model-training pipeline as an end-to-end system for integrating new AI models that satisfy specific departmental needs. The results from the retrospective contour QA confirm the utility of leveraging DL segmentations from TotalSegmentator both for providing automated delineations and for verifying contours for up to 104 different structures. By reporting on both Full DSC and Partial DSC metrics, the DL-ODA alerts users of contours showing poor agreement with DL segmentations and offers guidance as to whether the contours need to be revisited.

One drawback of the DL-ODA system is that it currently runs mainly on specific DL frameworks (nnU-Net and TotalSegmentator). However, the flexibility and generalizability of our workflow will allow for seamless incorporation of alternative models in future releases of DL-ODA. Given the rapid advancements of AI-based technologies in radiation oncology, new and improved AI segmentation platforms are constantly on the horizon, including Auto3DSeg from MONAI (44), which rivals the nnU-Net framework, and Segment Anything Model by Meta AI (45) which, like TotalSegmentator, specializes in full image segmentation. Another drawback is that the DL-ODA is an on-premises solution, requiring use of local computers and hospital networks. Latency issues or network outages would prevent users from using our system. One potential solution would be to adopt a cloud-based implementation. Although this would likely necessitate additional measures regarding patient data encryption, our system framework is already designed to accommodate any file-sharing protocol at any institution and a cloud-based solution could be adopted to scale up DL-ODA usage. In addition, DL-ODA is agnostic to patient demographic representations that are included in model training datasets. It is important for users to be aware of potential differences in clinical outcomes that may arise from deploying models that were trained using insufficient data representing marginalized communities. One possible solution is to train dedicated models specifically for these communities, utilizing the data augmentation tools built into our model-training pipeline for small datasets.

The DL-ODA is being prospectively evaluated by our clinic and we are in the process of gathering data regarding the employment of DL-ODA in clinical practice. Currently, the model training pipeline is run by two of our staff who are familiar with the source code whereas a more user-friendly interface is planned for the future. Ambient QA has been set up by one of our lab members to run every night, and it has been running without major downtime for over a year (any downtime thus far has been due to power outages and expired user accounts). The Ambient QA currently runs on one of our lab computers, though in the future we aim to host it on one of our institution’s servers maintained by our IT department. The On-Demand QA is based on the Eclipse Scripting API with a small graphical user interface that allows intuitive selection of tasks.

In conclusion, we have successfully developed, tested, and prospectively deployed DL-ODA in the clinic as an efficient, fully automated system that significantly improves RT planning tasks involving structure delineations and provides critical QA feedback for whole-body, heart, lung, and spine RT as well as gynecological and prostate brachytherapy. With the DL-ODA system, scripting, and models made publicly available to facilitate a more widespread adoption of AI technology, our efforts mark a major milestone in promoting accessibility of AI applications and in enabling rapid clinical translation.

## Data availability statement

The data analyzed in this study is subject to the following licenses/restrictions: The datasets used for this study are owned and maintained by our institution’s radiation oncology department. Requests to access these datasets should be directed to christian_guthier@dfci.harvard.edu.

## Ethics statement

The studies involving humans were approved by Dana-Farber/Harvard Cancer Center #20-328. The studies were conducted in accordance with the local legislation and institutional requirements. Written informed consent for participation was not required from the participants or the participants’ legal guardians/next of kin in accordance with the national legislation and institutional requirements.

## Author contributions

CK: Writing – original draft, Writing – review & editing. YY: Writing – review & editing. DB: Writing – review & editing. SQ: Writing – review & editing. DSB: Writing – review & editing. JB: Writing – review & editing. HA: Writing – review & editing. RM: Writing – review & editing. CG: Writing – review & editing.
